# Chloroplast thioredoxin systems: prospects for improving photosynthesis

**DOI:** 10.1098/rstb.2016.0474

**Published:** 2017-08-14

**Authors:** Lauri Nikkanen, Jouni Toivola, Manuel Guinea Diaz, Eevi Rintamäki

**Affiliations:** Molecular Plant Biology, Department of Biochemistry, University of Turku, 20014 Turku, Finland

**Keywords:** thioredoxin reductase, redox regulation, light reactions, Calvin–Benson cycle, NTRC, FTR

## Abstract

Thioredoxins (TRXs) are protein oxidoreductases that control the structure and function of cellular proteins by cleavage of a disulphide bond between the side chains of two cysteine residues. Oxidized thioredoxins are reactivated by thioredoxin reductases (TR) and a TR-dependent reduction of TRXs is called a thioredoxin system. Thiol-based redox regulation is an especially important mechanism to control chloroplast proteins involved in biogenesis, in regulation of light harvesting and distribution of light energy between photosystems, in photosynthetic carbon fixation and other biosynthetic pathways, and in stress responses of plants. Of the two plant plastid thioredoxin systems, the ferredoxin-dependent system relays reducing equivalents from photosystem I via ferredoxin and ferredoxin-thioredoxin reductase (FTR) to chloroplast proteins, while NADPH-dependent thioredoxin reductase (NTRC) forms a complete thioredoxin system including both reductase and thioredoxin domains in a single polypeptide. Chloroplast thioredoxins transmit environmental light signals to biochemical reactions, which allows fine tuning of photosynthetic processes in response to changing environmental conditions. In this paper we focus on the recent reports on specificity and networking of chloroplast thioredoxin systems and evaluate the prospect of improving photosynthetic performance by modifying the activity of thiol regulators in plants.

This article is part of the themed issue ‘Enhancing photosynthesis in crop plants: targets for improvement'.

## 1. Introduction

Photosynthesis comprises light-driven reactions in thylakoid membranes producing NADPH and ATP, and a CO_2_ fixation pathway storing the energy captured by light reactions into sugar phosphates. Photosynthesis end products are used to energize cell metabolism and to promote plant growth. In Nature, light intensity is constantly changing in plant growth habitats, including both seasonal alteration of daily light period and daily fluctuation of light intensity due to cloudiness and other environmental factors. Optimization of photosynthetic production under fluctuating light conditions needs strict balancing of the absorption of light energy by the photosynthetic machinery with the energy-consuming reactions of chloroplast metabolism. Photosynthetic reactions are also linked with the protective machinery that eliminates or prevents the generation of harmful reactive oxygen species (ROS). The regulatory mechanisms to balance light capture, consumption of light energy and inductive protective machinery include non-photochemical quenching (NPQ), photosynthetic control of electron flow between photosystem II (PSII) and photosystem I (PSI), state transitions, cyclic electron flow, light activation of photosynthetic enzymes both in light reactions and carbon fixation, and induction of antioxidant systems. Recently, the regulatory proteins called thioredoxins (TRXs) have been suggested to control a number of the mechanisms balancing photosynthetic reactions in chloroplasts.

TRXs are protein oxidoreductases with a redox active dithiol/disulphide motif in the active site. In the reduced state they alter the thiol-redox status of cellular proteins by reducing a disulphide bond in target proteins via a bimolecular nucleophilic substitution reaction. TRXs that become oxidized in the reaction are reactivated by thioredoxin reductases (TR). TR together with TRX form a thioredoxin system. Thioredoxin systems in plants are versatile including two TRs, one dependent on ferredoxin (FTR) and the other on NADPH (NTR) as reducing power, respectively, and multiple types of TRXs (h, o, f, m, x, y, z, and several thioredoxin-like proteins) (reviewed in [1–5]). A high number of TRXs are localized to plant chloroplasts; this underlines the impact of redox-regulation of chloroplast proteins. The ferredoxin-TRX system has been directly linked with plant production since it was originally shown to activate the enzymes of the Calvin–Benson cycle in algal and plant plastids upon illumination (see [5]). The discovery of the chloroplast NADPH-dependent thioredoxin system (NTRC) [6] and novel TRX types [7] has indicated that in addition to light, chloroplast TRX systems also control chloroplast development, respond to fluctuations in light intensity, transfer signals between chloroplast compartments and are involved in antioxidant networks (reviewed in [2–4,8–10]). Another intriguing issue is that some chloroplast proteins are targets of several TRXs. For example, NTRC, TRXf, and TRXm interact with the same biosynthetic enzymes, and the antioxidative system based on peroxiredoxins is maintained by TRXx, y, and NTRC [11–17]. The diversity and partial redundancy of chloroplast TRX systems may enable a rapid response of chloroplast metabolism to ever changing environmental conditions, raising plant fitness under natural growth conditions.

Here, we review the prospect of improving photosynthetic performance by modifying the activity of chloroplast TRX systems based on the recent reports on the interaction, networking and overlapping functions of TRXs.

## 2. Chloroplast thioredoxin systems

Comprehensive reviews on chloroplast TRX systems have been recently published [1–5,10]; hence only an overview is presented here. Chloroplast TRXs are activated by two TRs, FTR and NTRC. The former uses photoreduced ferredoxin and the latter NADPH to activate the TRXs. FTR is a heterodimeric iron-sulphur protein consisting of a catalytic and a variable subunit encoded by a single copy and two genes in *Arabidopsis*, respectively [18–20]. FTR has a binding site both for ferredoxin and TRX. A single nuclear gene of *Arabidopsis* encodes the NTRC enzyme that has an atypical structure consisting of a NADPH-dependent TR domain (NTR) fused to a TRX domain [12]. Thus the NTRC polypeptide forms a complete NADPH-dependent TRX system in the chloroplast. In photosynthetic cells the FTR-dependent system is active in light when PSI reduces ferredoxin, whereas the NTRC-dependent TRX system is active both in light and dark conditions because of the production of NADPH by the oxidative pentose phosphate pathway in darkness.

Five types of low-molecular weight TRXs (f, m, y, x, z) are localized to the chloroplast, of which TRXf, TRXm and TRXy are present as two, four and two isoforms, respectively [7,21]. CDSP32, six ACHT-type and two WCRKC-type proteins belong to the chloroplast TRX-like proteins having an atypical redox-active motif in the active site of the regulatory protein [7,21]. In addition to the soluble TRXs, chloroplasts contain thylakoid-bound TRX-like proteins, which control the redox status of thylakoid and lumenal proteins. Internal thylakoid protein CcdA mediates redox equivalents from stromal TRXs to the TRX-like transmembrane protein HCF164 that has a redox-active site in the lumenal side of the thylakoid membrane [8]. HCF164 has been suggested to regulate the redox status of lumenal proteins [8,10]. Suppressor of quenching (SOQ1) and lumen thiol oxidoreductase 1 (LTO1) are other thylakoid TRX-like proteins with a redox-active motif in the lumenal side of thylakoids [22,23]. Additionally several uncharacterized TRX-like proteins with unknown function are predicted to be localized to thylakoid membranes.

The amount of TRXs, like the regulatory proteins in general, is low in chloroplasts. The amount of the substrates of TRXs (e.g. the redox-activated enzymes in the Calvin–Benson cycle, 2-Cys peroxiredoxins) has been estimated to be 10–100 times higher than the amount of TRXs in chloroplast [24,25]. Belin *et al*. [21] have analysed the relative expression level of the genes encoding TRXs and TRs in different *Arabidopsis* organs. Among the chloroplast TRX genes the expression of TRXm1, 2, 4 isoforms, CDSP32 and TRXf1 isoform was highest in photosynthetic tissues, followed by TRXx and TRXy2. The expression of the other chloroplast TRX genes was modest or low in photosynthetic tissues, or occurred at a specific developmental stage like ACHT4 in senescing leaves [21]. The expression level of the genes encoding the TR was 2–4 times lower than the level of highly-expressed chloroplast TRXs, FTR being more expressed than NTRC [21]. The transcript levels of TRXs correlates with the content of proteins in leaf tissues, TRXm1, 2, 4 isoforms being the most abundant followed by CDSP32 [24,25]. The amount of TRXf1 protein has been reported to be slightly lower [24,25] or higher [16] than TRXm isoforms. NTRC protein almost equals the content of abundant chloroplast TRXs in photosynthetic cells [25], whereas to our knowledge the abundance of FTR protein has not been reported.

## 3. Thioredoxin networks in chloroplast

Purified and recombinant TRX and TR proteins, TRX-affinity chromatography, the yeast-2-hybrid test (Y2H), bimolecular fluorescence complementation (BiFC) assays together with knockout mutants and overexpression lines have been used to investigate which TR mediates the reducing power of chloroplast TRXs. Originally Buchanan *et al.* reported the reduction of TRXf and -m isoforms by the light-activated FTR system (see the history of the chloroplast TRX system in [5]) and this discovery has been confirmed by several *in vitro* and *in vivo* tests [17,26,27]. FTR can also reduce TRXy and x isoforms *in vitro* [26], whereas contradictory observations have been published about the reductant for TRXz. Bohrer *et al*. [26] reported the inability of FTR and NTRC to reduce TRXz but that the reduced TRXf, m, x and y isoforms were able to activate TRXz. In contrast, Yoshida & Hisabori [27] reported that NTRC is a primary reductant of TRXz *in vitro*, while BiFC tests failed to show interactions between NTRC and TRXz [17].

Upon the discovery of NTRC [6] it was presumed that NTRC forms an independent TRX system functionally separated from the FTR system. Recently, however, we reported that transgenic *Arabidopsis* lines overexpressing a mutated NTRC with either an inactivated NTR or TRX domain in *ntrc* mutant background, showed partial recovery of the *ntrc* phenotype, suggesting interaction between the NTRC and FTR systems [28]. Subsequent BiFC assays indeed showed that NTRC can interact with several soluble TRXs (f1, m1 and m3, y1 and x) *in vivo* in the chloroplast [17]. Furthermore, the content of reduced TRXf form was significantly higher under all studied light intensities in transgenic lines overexpressing wild-type NTRC (OE-NTRC) and under low light in transgenic lines with an inactive TRX domain of NTRC [17], indicating that NTRC is capable of transferring reducing equivalents to TRXf *in vivo*. Our observation contradicts *in vitro* assays of electron donation between NTRC and TRXs [26,27]. The midpoint redox potential (E_m,7.0_) of NTRC was reported to be too high (−274 mV) [27] for it to reduce TRXf, m, x and y isoforms (E_m,7.0_ between −290 and −320 mV in [13]). Nevertheless, it has been undeniably shown that NTRC is a primary reductant for chloroplast 2-Cys peroxiredoxins (2-Cys-PRX) [12,29], although a low midpoint redox potential of −315 mV has been measured for 2-Cys-PRXs [30,31].

**Figure 1.**
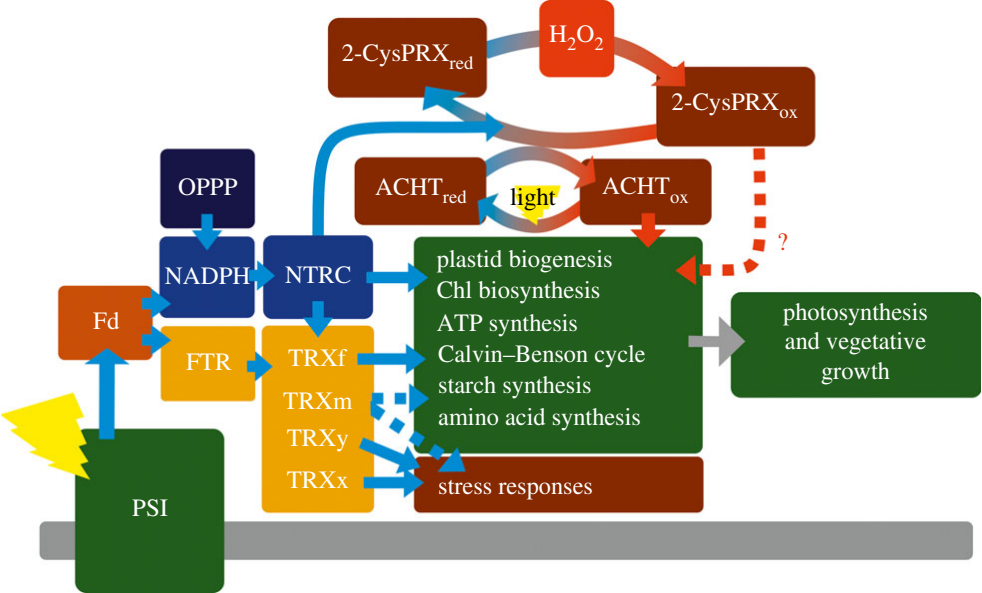
Proposed model for regulation of photosynthesis and stress reactions by the chloroplast thioredoxin network. Light activates the FTR-dependent TRX system, while NTRC can be activated both by light and by NADPH produced in the oxidative pentose phosphate pathway (OPPP). NTRC, TRXf and to a lesser extent TRXm are involved in the activation of enzymes in biosynthetic pathways that promote photosynthetic productivity and growth in plants. TRXy and x and probably also TRXm control the proteins involved in stress reactions. Hydrogen peroxide, 2-Cys PRXs and ACHT form an oxidation loop to the TRX network that controls the redox homeostasis of chloroplast metabolism under fluctuating light conditions and light–dark transitions. Enhancement of NTRC activity promotes growth by increasing biosynthetic activity of the chloroplast and decreasing the accumulation of oxidized 2-Cys PRXs. Reducing signals are depicted by blue arrows and oxidizing signals by red arrows. For details, see §3.

All the techniques used to investigate the interactions between the components of TRX systems are prone to false interpretation. *In vitro* conditions do not always correspond to *in vivo* circumstances. Purified TRXs and NTRC have a tendency to form oligomeric aggregates [32–34] that may affect the content of functional protein in *in vitro* assays. In BiFC assays, the tested proteins are overexpressed, which may facilitate the interactions and result in false positives. Nevertheless, the recently reported double mutants of the TRX systems support the idea that FTR and NTRC systems form a functional overlapping redox network that allows the chloroplast metabolism to flexibly respond to environmental changes in the growth habitat. The TRXf isoforms have been regarded as primary activators of redox-regulated enzymes in the Calvin–Benson cycle, but knockout of both genes encoding the two isoforms did not compromise the growth of *Arabidopsis*, and activation of the enzymes of the Calvin–Benson cycle in the light was only slightly impaired [16,35]. Knockout of NTRC, however, significantly compromised the development, photosynthetic activity and growth of *Arabidopsis* leaves [12,36], but the phenotype of the *ntrc trxf1* double mutant was even more drastically affected [15], suggesting that NTRC and TRXf are involved in the regulation of the same chloroplast reactions. They either function independently in parallel or sequentially by reduction of TRXf by both FTR and NTRC [17] (figure 1).

Much less is known about the reduction of chloroplast TRX-like proteins. ACHT1 protein was shown to be reduced in the light but the electron donor was not identified [37]. TRXm has been demonstrated to donate electrons to thylakoid HCF164 protein via reduction of CcdA [8].

Reduction of ferredoxin and NADP^+^ in light reactions links the TRX systems to the regulation of chloroplast processes in the dark–light transition. However, what is less well-characterized is how the reduced state of TRXs and their target proteins is maintained under continuous and fluctuating light. Molecular oxygen has been shown to oxidize the redox-regulated enzymes [13], and because of the evolution of oxygen by light reactions, a continuous supply of reducing equivalents from the TRX systems is needed to maintain the activation state of enzymes in illuminated chloroplasts. However, in addition to the reducing step of redox regulation, also the oxidation cycle of TRX systems may be strictly controlled under fluctuating light [2]. Danon and his colleagues [37,38] have described an oxidizing loop for a TRX system employing chloroplast 2-Cys-PRXs that controls starch synthesis under fluctuating light and in light–dark transition. 2-Cys-PRXs are abundant chloroplast proteins that are oxidized by a reaction with hydrogen peroxide [30]. Oxidized 2-Cys-PRXs are primarily reactivated by NTRC [12,17], but they can also take electrons from TRX-like proteins, ACHT1 and ACHT4 [37,38]. Oxidized ACHT4, in turn inactivated ADP-glucose pyrophosphorylase (AGPase), a redox-regulated enzyme in starch synthesis, thus slowing down starch synthesis in the light–dark transition and under fluctuating light [38]. We have shown that the high accumulation of oxidized 2-Cys-PRXs in *Arabidopsis* leaves lacking functional NTRC correlates with the oxidation of redox-regulated Calvin–Benson enzymes [17], suggesting that oxidized 2-Cys-PRXs may act as a general oxidizing loop for redox-regulated enzymes in the chloroplast [39] (figure 1).

## 4. Thioredoxin-dependent regulation of photosynthesis

Potential target proteins regulated by TRXs cover all the essential processes in chloroplasts, including biogenesis of plastids, gene expression, translation, photosynthesis, antioxidant and stress reactions and biosynthetic metabolism [2–4,13,40], underlining a vital role of TRXs in plant chloroplasts. In this review we shall focus on the TRX-dependent regulation of photosynthetic light reactions and carbon fixation, and evaluate the prospects for improving photosynthetic productivity by modification of chloroplast TRX systems.

TRXs regulate a number of proteins directly or indirectly linked to photosynthetic reactions, including the biogenesis of chloroplasts through activation of the redox-controlled enzymes in the tetrapyrrole pathway that synthethizes chlorophyll and haem [41]. Additionally, the redox-active Cys residues in the γ-subunit of ATP synthase are rapidly reduced in the light and oxidized in darkness, so forming an on/off regulatory loop for ATP production in chloroplasts [42]. Previously the FTR system had been regarded as the primary reductant of the γ-subunit upon illumination [13], but recent results have provided both biochemical and biophysical evidence that also identifies NTRC as a key regulator of the ATP synthase, with a non-redundant role in reducing the γ-subunit under low irradiance [17,43,44]. Thiol regulation of the other primary components in light reactions has not been reported but the mechanisms controlling light harvesting and distribution of light energy in thylakoid photosystems are prone to redox regulation by TRXs. STN7 kinase is inactivated by TRX that switches off state transition in plants transferred to high light conditions [45,46] and NPQ is stimulated in the *soq1* knockout mutant that lacks a thylakoid-bound TRX-like protein [23]. The latter finding is supported by the observation that dithiothreitol treatment decreased NPQ yield in *Arabidopsis* leaves [44]. Thioredoxins have also been reported to regulate PGR5/PGRL1-dependent cyclic electron flow [47,48].

In chloroplast carbon metabolism, light-induced activation of the enzymes in the Calvin–Benson cycle is the best-characterized action of chloroplast TRXs. The TRX system activates the following Calvin–Benson cycle enzymes in the dark–light transition by reduction of redox-active Cys residues (see [5]): glyceraldehyde-3-phosphate dehydrogenase (GAPDH), fructose-bisphosphatase (FBPase), sedoheptulose-bisphosphatase (SBPase) and phosphoribulokinase (PRK). Rubisco is also indirectly regulated by thioredoxin via activation of Rubisco activase. Also the key enzymes in the pathways closely linked to photosynthesis (AGPase in starch synthesis and NADP-malate dehydrogenase (NADP-MDH) in the malate oxaloacetate shuttle) are regulated by TRXs.

The specificity of TRX isoforms in the regulation of carbon fixation has recently been widely debated. The double (*trxf1 trxf2*) [16,35] and triple (*trxm1 trxm2 trxm4*) [49] knockout mutants support the original *in vitro* observations that TRXf and m isoforms are involved in the regulation of photosynthetic enzymes. Furthermore, NTRC is shown to interact with the PRK and FBPase in BiFC tests and the activation levels of these enzymes were higher in illuminated *Arabidopsis* leaves overexpressing the NTRC gene [17], whereas the light-dependent redox-activation of FBPase was more impaired in a *ntrc trxf1* double mutant than in a single *ntrc* or *trxf1* mutant [15]. These observations suggest that either NTRC directly regulates the enzymes in the Calvin–Benson cycle or that NTRC is involved in the activation of TRXf which in turn controls the activity of these enzymes. However, the conclusions drawn from the knockout mutants of TRXs should be viewed with caution, because apart from a direct effect of a missing TRX on the enzymes, the overall redox state of the chloroplast may be altered in the mutants. Especially if the mutation modifies the oxidation loop in the reversible redox-regulation cycle of TRX targets (figure 1), the amount of redox-activated enzymes may be lower in mutants than in wild-type plants. Knockout of NTRC [29] and TRXm1, 2 and 4 isoforms [50] increases production of ROS. Elevated oxidative stress induces the accumulation of oxidized components, including 2-Cys-PRXs in the chloroplast [17,29] that in turn may remodify the redox state of chloroplast enzymes *in vivo* as described in [38] (figure 1). In this case the lower level of activated enzymes is not directly due to the loss of a knockout TRX but to the elevated amount of oxidant(s) in chloroplast.

## 5. Improving photosynthesis by modification of the chloroplast thioredoxin network

Among the chloroplast TRXs, TRXf and NTRC have been specifically linked to the activation of photosynthesis and biosynthetic pathways in the chloroplast by several publications reviewed in §3 (figure 1). They control ATP synthase and the redox-activated enzymes in the tetrapyrrole pathway producing haem and chlorophylls, the shikimate pathways synthesizing aromatic amino acids, the Calvin–Benson cycle and starch synthesis (reviewed in [2,3]). NTRC also functions as an antioxidant in the chloroplast by primarily activating the hydrogen peroxide scavengers 2-Cys PRXs. TRXf and the TRX domain of NTRC have very similar electrostatic surface charges, suggesting that they can have overlapping targets in chloroplasts [28]. Thus it is presumable that boosting the activity of these TRXs may promote biosynthetic activities in chloroplasts and enhance plant growth. Indeed, the overexpression of TRXf in tobacco (OE-TRXf) [51] and NTRC in *Arabidopsis* (OE-NTRC) [17,28] stimulated the biomass production of plants and increased leaf size (OE-NTRC) or specific leaf mass (OE-TRXf). It is remarkable that the overexpression of TRXm did not stimulate growth of tobacco [51]. The overexpressed protein content was 10- to 20-fold (OE-NTRC) and 20- to 200-fold higher (OE-TRXf) than in wild-type leaves, which roughly equals the concentration of TRX targets in the chloroplast [24]. Overexpression of TRXf did not increase the steady-state photosynthesis of tobacco but the accumulation of starch and sugars (glucose, fructose, sucrose) in the leaves had risen significantly [51]. In contrast, overexpression of NTRC increased photosynthetic activity in *Arabidopsis* [17]. The quantum yield of PSI was increased under light intensities lower than growth light and NPQ decreased under high light, suggesting that OE-NTRC plants can efficiently use absorbed light energy in the chloroplast. Steady-state CO_2_ fixation was about 20% higher than in wild-type and the changes in the photosynthetic parameters correlated with the higher accumulation of reduced forms of redox-controlled enzymes. Accumulation of fully-oxidized 2-Cys PRXs was eliminated both in darkness and light, suggesting that also the oxidation loop of redox-regulated enzymes was modified in OE-NTRC plants. Overexpression of NTRC did not cause any visible reduction of growth under optimal growth conditions, but seed production was about 20% lower than in wild-type (Lauri Nikkanen 2016, unpublished), suggesting that high NTRC content favours vegetative growth.

OE-NTRC and OE-TRXf plants have a crucial difference that may affect the phenotype of the plants. NTRC represents a fully active TRX system including both the reductase activity and TRX activity in a single protein. Thus the activity of NTRC in overexpression plants depends only on the availability of NADPH in the chloroplasts. We have shown that overexpression of NTRC also increases the amount of reduced TRXf *in vivo* and this reduction depends on the reductase domain of NTRC [17]. Thus, boosting the NTRC system also stimulates the regulation of TRXf targets, which may partly explain the overall positive effect of OE-NTRC on photosynthesis. However, high NTRC content also decreased the accumulation of oxidized 2-Cys PRXs under all light conditions [17]. If, as suggested by Eliyahu *et al*. [38], oxidized 2-Cys PRXs are involved in the transient modification of redox-regulated proteins, the extra amount of NTRC may alter the responses of photosynthesis to fluctuating light conditions. In OE-TRXf tobacco plants, only the amount of TRXf was raised, while the leaves still had endogenous content of TRs. The amount of active TRXf in tobacco leaves overexpressing TRXf was not estimated [51] but the low endogenous amount of TR may limit the effective utilization of extra TRXf in chloroplasts. We have constructed a chimeric NTRC, in which the TRX domain of NTRC has been replaced by the TRXf sequence (Jouni Toivola 2016, unpublished). NTRC-TRXf would be reduced by NADPH *in vivo*, like NTRC, and thus the overexpression of this chimeric NTRC-TRXf may elevate the active TRXf content in chloroplasts over that found in OE-TRXf tobacco plants. We have transferred the NTRC-TRXf construct to *Arabidopsis* and the screening of transgenic lines are ongoing.

## 6. Future prospects

The present experimental data emphasize that elevated activity of specific chloroplast TRXs (NTRC, TRXf) promotes vegetative growth and sugar production in plants under optimal greenhouse conditions. The growth stimulation is linked to photosynthesis with improved capacity to use light energy (OE-NTRC) and increased production of starch and soluble sugars in leaves (OE-TRXf). These modifications of photosynthetic activity and sugar production may improve the potential of plants for production of biofuels or other sugar-based valuable compounds. Further studies are needed to evaluate whether the increased activity of NTRC and/or TRXf promotes the growth of other plant species and whether this modification does or does not compromise the growth under stress conditions, including fluctuating light. The other chloroplast TRXs, especially TRXm, TRXy, TRXx, and CDP32 have been shown to regulate chloroplast proteins under stress conditions [11,52–54]. It remains to be elucidated whether the elevated activity of these TRXs will improve plant tolerance to stresses.

## Data accessibility

This article has no data.

## References

[1] Balsera M, Uberegui E, Schürmann P, Buchanan BB 2014 Evolutionary development of redox regulation in chloroplasts. Antioxid. Redox Signal. 21, 1327–1355. (10.1089/ars.2013.5817) 24483204

[2] Nikkanen L, Rintamaki E 2014 Thioredoxin-dependent regulatory networks in chloroplasts under fluctuating light conditions. Phil. Trans. R. Soc. B 369, 20130224 (10.1098/rstb.2013.0224) 24591711 PMC3949389

[3] Geigenberger P, Fernie AR 2014 Metabolic control of redox and redox control of metabolism in plants. Antioxid. Redox Signal. 21, 1389–1421. (10.1089/ars.2014.6018) 24960279 PMC4158967

[4] Rouhier N, Cerveau D, Couturier J, Reichheld JP, Rey P 2015 Involvement of thiol-based mechanisms in plant development. Biochim. Biophys. Acta 1850, 1479–1496. (10.1016/j.bbagen.2015.01.023) 25676896

[5] Buchanan BB 2016 The path to thioredoxin and redox regulation in chloroplasts. Annu. Rev. Plant Biol. 67, 1–24. (10.1146/annurev-arplant-043015-111949) 27128465

[6] Serrato AJ, Pérez-Ruiz JM, Spinola MC, Cejudo FJ 2004 A novel NADPH thioredoxin reductase, localized in the chloroplast, which deficiency causes hypersensitivity to abiotic stress in *Arabidopsis thaliana*. J. Biol. Chem. 279, 43 821–43 827. (10.1074/jbc.M404696200) 15292215

[7] Meyer Y, Siala W, Bashandy T, Riondet C, Vignols F, Reichheld JP 2008 Glutaredoxins and thioredoxins in plants. Biochim. Biophys. Acta 1783, 589–600. (10.1016/j.bbamcr.2007.10.017) 18047840

[8] Motohashi K, Hisabori T 2010 CcdA is a thylakoid membrane protein required for the transfer of reducing equivalents from stroma to thylakoid lumen in the higher plant chloroplast. Antioxid. Redox Signal. 13, 1169–1176. (10.1089/ars.2010.3138) 20214498

[9] Bölter B, Soll J, Schwenkert S 2015 Redox meets protein trafficking. Biochim. Biophys. Acta 1847, 949–956. (10.1016/j.bbabio.2015.01.010) 25626173

[10] Kang ZH, Wang GX 2016 Redox regulation in the thylakoid lumen. J. Plant Physiol. 192, 28–37. (10.1016/j.jplph.2015.12.012) 26812087

[11] Collin V, Issakidis-Bourguet E, Marchand C, Hirasawa M, Lancelin JM, Knaff DB, Miginiac-Maslow M 2003 The *Arabidopsis* plastidial thioredoxins: new functions and new insights into specificity. J. Biol. Chem. 278, 23 747–23 752. (10.1074/jbc.M302077200) 12707279

[12] Pérez-Ruiz JM, Spinola MC, Kirchsteiger K, Moreno J, Sahrawy M, Cejudo FJ 2006 Rice NTRC is a high-efficiency redox system for chloroplast protection against oxidative damage. Plant Cell 18, 2356–2368. (10.1105/tpc.106.041541) 16891402 PMC1560923

[13] Schürmann P, Buchanan BB 2008 The ferredoxin/thioredoxin system of oxygenic photosynthesis. Antioxid. Redox Signal. 10, 1235–1274. (10.1089/ars.2007.1931) 18377232

[14] Ikegami A, Yoshimura N, Motohashi K, Takahashi S, Romano PG, Hisabori T, Takamiya K, Masuda T 2007 The CHLI1 subunit of *Arabidopsis thaliana* magnesium chelatase is a target protein of the chloroplast thioredoxin. J. Biol. Chem. 282, 19 282–19 291. (10.1074/jbc.M703324200) 17472958

[15] Thormählen I, Meitzel T, Groysman J, Ochsner AB, von Roepenack-Lahaye E, Naranjo B, Cejudo FJ, Geigenberger P 2015 Thioredoxin f1 and NADPH-dependent thioredoxin reductase C have overlapping functions in regulating photosynthetic metabolism and plant growth in response to varying light conditions. Plant Physiol. 3, 1766–1786. (10.1104/pp.15.01122) PMC4634086 26338951

[16] Yoshida K, Hara S, Hisabori T 2015 Thioredoxin selectivity for thiol-based redox regulation of target proteins in chloroplasts. J. Biol. Chem. 32, 19540 (10.1074/jbc.A115.647545) PMC4528118 26254269

[17] Nikkanen L, Toivola J, Rintamäki E 2016 Crosstalk between chloroplast thioredoxin systems in regulation of photosynthesis. Plant Cell Environ. 39, 1691–1705. (10.1111/pce.12718) 26831830

[18] Dai S, Schwendtmayer C, Peter Schurmann P, Ramaswamy S, Eklund H 2000 Redox signaling in chloroplasts: cleavage of disulphides by an iron-sulfur cluster. Science 287, 655–658. (10.1126/science.287.5453.655) 10649999

[19] Balsera M, Uberegui E, Susanti D, Schmitz RA, Mukhopadhyay B, Schürmann P, Buchanan BB 2013 Ferredoxin:thioredoxin reductase (FTR) links the regulation of photosynthesis to deeply rooted bacteria. Planta 237, 619–635. (10.1007/s00425-012-1803-y) 23223880

[20] Wang P, Liu J, Liu B, Da Q, Feng D, Su J, Zhang Y, Wang J, Wang HB 2014 Ferredoxin:thioredoxin reductase is required for proper chloroplast development and is involved in the regulation of plastid gene expression in *Arabidopsis thaliana*. Mol. Plant 7, 1586–1590. (10.1093/mp/ssu069) 24890758

[21] Belin C, Bashandy T, Cela J, Delorme-Hinoux V, Riondet C, Reichheld JP 2015 A comprehensive study of thiol reduction gene expression under stress conditions in *Arabidopsis thaliana*. Plant Cell Environ. 38, 299–314. (10.1111/pce.12276) 24428628

[22] Karamoko M, Cline S, Redding K, Ruiz N, Hamel PP 2011 Lumen thiol oxidoreductase1, a disulfide bond-forming catalyst, is required for the assembly of photosystem II in *Arabidopsis*. Plant Cell 23, 4462–4475. (10.1105/tpc.111.089680) 22209765 PMC3269877

[23] Brooks MD, Sylak-Glassman EJ, Fleming GR, Niyogi KK 2013 A thioredoxin-like/beta-propeller protein maintains the efficiency of light harvesting in *Arabidopsis*. Proc. Natl Acad. Sci. USA 110, E2733–E274010. (10.1073/pnas.1305443110) 23818601 PMC3718131

[24] Peltier JB, Cai Y, Sun Q, Zabrouskov V, Giacomelli L, Rudella A, Ytterberg AJ, Rutschow H, van Wijk KJ 2006 The oligomeric stromal proteome of *Arabidopsis thaliana* chloroplasts. Mol. Cell. Proteomics 5, 114–133. (10.1074/mcp.M500180-MCP200) 16207701

[25] König J, Muthuramalingam M, Dietz K 2012 Mechanisms and dynamics in the thiol/disulfide redox regulatory network: transmitters, sensors and targets. Curr. Opin. Plant Biol. 15, 261–268. (10.1016/j.pbi.2011.12.002) 22226570

[26] Bohrer AS, Massot V, Innocenti G, Reichheld JP, Issakidis-Bourguet E, Vanacker H 2012 New insights into the reduction systems of plastidial thioredoxins point out the unique properties of thioredoxin z from *Arabidopsis*. J. Exp. Bot. 63, 6315–6323. (10.1093/jxb/ers283) 23096001

[27] Yoshida K, Hisabori T 2016 Two distinct redox cascades cooperatively regulate chloroplast functions and sustain plant viability. Proc. Natl Acad. Sci. USA 113, E3967–E3976. (10.1073/pnas.1604101113) 27335455 PMC4941451

[28] Toivola J, Nikkanen L, Dahlström KM, Salminen TA, Lepistö A, Vignols F, Rintamäki E 2013 Overexpression of chloroplast NADPH-dependent thioredoxin reductase in *Arabidopsis* enhances leaf growth and elucidates *in vivo* function of reductase and thioredoxin domains. Front. Plant Sci. 4, 389 (10.3389/fpls.2013.00389) 24115951 PMC3792407

[29] Pulido P et al. 2010 Functional analysis of the pathways for 2-Cys peroxiredoxin reduction in *Arabidopsis thaliana* chloroplasts. J. Exp. Bot. 61, 4043–4054. (10.1093/jxb/erq218) 20616155 PMC2935875

[30] Dietz KJ 2003 Plant peroxiredoxins. Annu. Rev. Plant Biol. 54, 93–107. (10.1146/annurev.arplant.54.031902.134934) 14502986

[31] Chibani K, Tarrago L, Schürmann P, Jacquot JP, Rouhier N 2011 Biochemical properties of poplar thioredoxin z. FEBS Lett. 585, 1077–1081. (10.1016/j.febslet.2011.03.006) 21385584

[32] Pérez-Ruiz JM, González M, Spínola MC, Sandalio LM, Cejudo FJ 2009 The quaternary structure of NADPH thioredoxin reductase C is redox-sensitive. Mol. Plant 2, 457–467. (10.1093/mp/ssp011) 19825629

[33] Sanz-Barrio R, Fernández-San Millán A, Carballeda J, Corral-Martínez P, Seguí-Simarro JM, Farran I 2012 Chaperone-like properties of tobacco plastid thioredoxins f and m. J. Exp. Bot. 63, 365–379. (10.1093/jxb/err282) 21948853 PMC3245471

[34] Wulff RP, Lundqvist J, Rutsdottir G, Hansson A, Stenbaek A, Elmlund D, Elmlund H, Jensen PE, Hansson M 2011 The activity of barley NADPH-dependent thioredoxin reductase C is independent of the oligomeric state of the protein: tetrameric structure determined by cryo-electron microscopy. Biochemistry 50, 3713–3723. (10.1021/bi200058a) 21456578

[35] Naranjo B, Diaz-Espejo A, Lindahl M, Cejudo FJ 2016 Type-f thioredoxins have a role in the short-term activation of carbon metabolism and their loss affects growth under short-day conditions in *Arabidopsis thaliana*. J. Exp. Bot. 67, 1951–1964. (10.1093/jxb/erw017) 26842981 PMC4783373

[36] Lepistö A, Kangasjärvi S, Luomala EM, Brader G, Sipari N, Keränen M, Keinänen M, Rintamäki E 2009 Chloroplast NADPH-thioredoxin reductase interacts with photoperiodic development in *Arabidopsis*. Plant Physiol. 149, 1261–1276. (10.1104/pp.108.133777) 19151130 PMC2649390

[37] Dangoor I, Peled-Zehavi H, Wittenberg G, Danon A 2012 A chloroplast light-regulated oxidative sensor for moderate light intensity in *Arabidopsis*. Plant Cell 24, 1894–1906. (10.1105/tpc.112.097139) 22570442 PMC3442576

[38] Eliyahu E, Rog I, Inbal D, Danon A 2015 ACHT4-driven oxidation of APS1 attenuates starch synthesis under low light intensity in *Arabidopsis* plants. Proc. Natl Acad. Sci. USA 112, 12 876–12 881. (10.1073/pnas.1515513112) PMC4611610 26424450

[39] Caporaletti D, D'Alessio AC, Rodriguez-Suarez RJ, Senn AM, Duek PD, Wolosiuk RA 2007 Non-reductive modulation of chloroplast fructose-1,6-bisphosphatase by 2-Cys peroxiredoxin. Biochem. Biophys. Res. Commun. 355, 722–727. (10.1016/j.bbrc.2007.02.013) 17307139

[40] Gelhaye E, Rouhier N, Navrot N, Jacquot JP 2005 The plant thioredoxin system. Cell. Mol. Life Sci. 62, 24–35. (10.1007/s00018-004-4296-4) 15619004 PMC11924577

[41] Brzezowski P, Richter AS, Grimm B 2015 Regulation and function of tetrapyrrole biosynthesis in plants and algae. Biochim. Biophys. Acta 1847, 968–985. (10.1016/j.bbabio.2015.05.007) 25979235

[42] Hisabori T, Sunamura EI, Kim Y, Konno H 2013 The chloroplast ATP synthase features the characteristic redox regulation machinery. Antioxid. Redox Signal. 19, 1846–1854. (10.1089/ars.2012.5044) 23145525 PMC3837435

[43] Carrillo LR, Froehlich JE, Cruz JA, Savage LJ, Kramer DM 2016 Multi-level regulation of the chloroplast ATP synthase: the chloroplast NADPH thioredoxin reductase C (NTRC) is required for redox modulation specifically under low irradiance. Plant J. 87, 654–663. (10.1111/tpj.13226) 27233821

[44] Naranjo B, Mignée C, Krieger-Liszkay A, Hornero-Méndez D, Gallardo-Guerrero L, Cejudo FJ, Lindahl M 2016 The chloroplast NADPH thioredoxin reductase C, NTRC, controls non-photochemical quenching of light energy and photosynthetic electron transport in *Arabidopsis*. Plant Cell Environ. 39, 804–822. (10.1111/pce.12652) 26476233

[45] Rintamäki E, Martinsuo P, Pursiheimo S, Aro EM 2000 Cooperative regulation of light-harvesting complex II phosphorylation via the plastoquinol and ferredoxin-thioredoxin system in chloroplasts. Proc. Natl Acad. Sci. USA 97, 11 644–11 649. (10.1073/pnas.180054297) PMC17254 11005828

[46] Tikkanen M, Aro EM 2014 Integrative regulatory network of plant thylakoid energy transduction. Trends Plant Sci. 19, 10–17. (10.1016/j.tplants.2013.09.003) 24120261

[47] Hertle AP, Blunder T, Wunder T, Pesaresi P, Pribil M, Armbruster U, Leister D 2013 PGRL1 is the elusive ferredoxin-plastoquinone reductase in photosynthetic cyclic electron flow. Mol. Cell 49, 511–523. (10.1016/j.molcel.2012.11.030) 23290914

[48] Strand DD, Fisher N, Davis GA, Kramer DM 2015 Redox regulation of the antimycin A sensitive pathway of cyclic electron flow around photosystem I in higher plant thylakoids. Biochim. Biophys. Acta 1857, 1–6. (10.1016/j.bbabio.2015.07.012) 26235611

[49] Okegawa Y, Motohashi K 2015 Chloroplastic thioredoxin m functions as major regulator of Calvin cycle enzymes during photosynthesis *in vivo*. Plant J. 84, 900–913. (10.1111/tpj.13049) 26468055

[50] Wang P et al. 2013 Evidence for a role of chloroplastic m-type thioredoxins in the biogenesis of photosystem II in *Arabidopsis*. Plant Physiol. 163, 1710–1728. (10.1104/pp.113.228353) 24151299 PMC3850194

[51] Sanz-Barrio R, Corral-Martinez P, Ancin M, Segui-Simarro JM, Farran I 2013 Overexpression of plastidial thioredoxin f leads to enhanced starch accumulation in tobacco leaves. Plant Biotechnol. J. 11, 618–627. (10.1111/pbi.12052) 23398733

[52] Collin V, Lamkemeyer P, Miginiac-Maslow M, Hirasawa M, Knaff DB, Dietz KJ, Issakidis-Bourguet E 2004 Characterization of plastidial thioredoxins from *Arabidopsis* belonging to the new y-type. Plant Physiol. 136, 4088–4095. (10.1104/pp.104.052233) 15531707 PMC535839

[53] Tarrago L, Laugier E, Zaffagnini M, Marchand CH, Le Maréchal P, Lemaire SD, Rey P 2010 Plant thioredoxin CDSP32 regenerates 1-cys methionine sulfoxide reductase B activity through the direct reduction of sulfenic acid. J. Biol. Chem. 285, 14 964–14 972. (10.1074/jbc.M110.108373) PMC2865344 20236937

[54] Rey P, Sanz-Barrio R, Innocenti G, Ksas B, Courteille A, Rumeau D, Issakidis-Bourguet E, Farran I 2013 Overexpression of plastidial thioredoxins f and m differentially alters photosynthetic activity and response to oxidative stress in tobacco plants. Front. Plant Sci. 16, 390 (10.3389/fpls.2013.00390) PMC3797462 24137166

